# Supplementary material for: Tailoring the superradiant and subradiant nature of two coherently coupled quantum emitters
Source: Nat Commun. 2022 May 26;13:2962. doi: 10.1038/s41467-022-30672-2 (PMC9135760; doi:10.1038/s41467-022-30672-2)
Supplement: Supplementary file 1 — Supplementary Information [file 41467_2022_30672_MOESM1_ESM.pdf]

Supplementary Information for

Tailoring the superradiant and subradiant nature of two coherently coupled quantum emitters

J.-B. Trebbia[1,2], Q. Deplano[1,2], P. Tamarat[1,2] and B. Lounis[1,2]*

1- Univ Bordeaux, LP2N, F- 33405 Talence, France

2- Institut d'Optique & CNRS, LP2N, F-33405 Talence, France

* Corresponding author, brahim.lounis@u-bordeaux.fr

This Supporting Information file contains:

Supplementary Fig. 1 to 13
Supplementary Notes 1 and 2
References

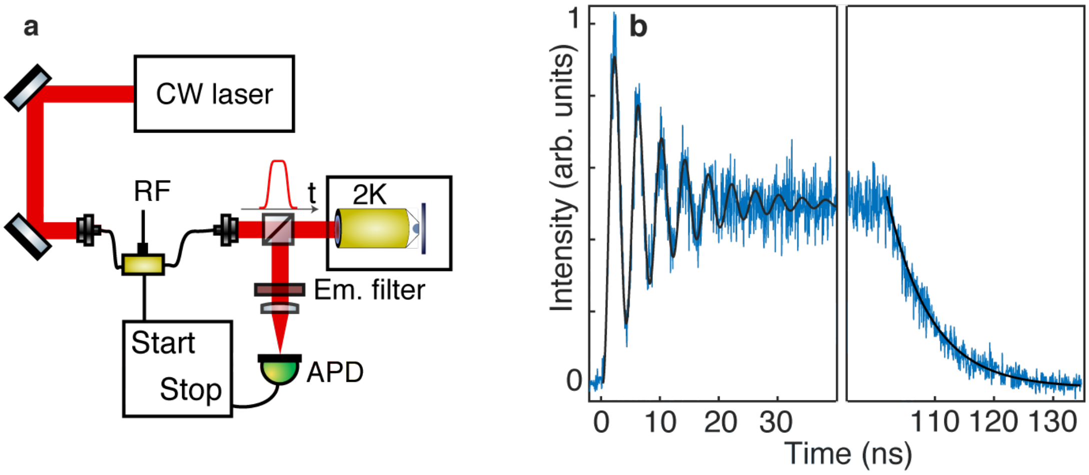

Supplementary Fig. 1: Single qubit rotation.
a, Sketch of the experimental setup: A CW dye laser, optically chopped with an optical modulator produces optical pulses with a duration of 100 ns and a rise time/fall time of 500 ps (10%-90%) at a repetition rate of 100 kHz. The laser frequency is locked on a wavemeter with a digital PID to the frequency of a single emitter ZPL within 10 MHz. An avalanche photodiode combined with a start-stop acquisition card synchronized with the excitation pulses records the arrival times of the red-shifted fluorescence photons stemming from the molecule. **b**, Temporal evolution of the fluorescence signal from an isolated single DBATT molecule upon pulsed resonant excitation (Integration time 25 min, bin width 64 ps). The fluorescence background stemming from out-of-focus molecules has been subtracted and the fluorescence signal is normalized to 1/2 at long times in order to mimic the evolution of the excited state population. The black curve is a theoretical simulation of the excited state population, which reproduces the damped Rabi oscillations following the leading edge of the excitation pulse, with a Rabi angular frequency 11.7 γ_0 and an optical coherence lifetime $T_2 = 2T_1$. The excited state lifetime $T_1 = (7.39 \pm 0.02)$ ns is deduced from the exponential decay following the trailing edge of the pulse.

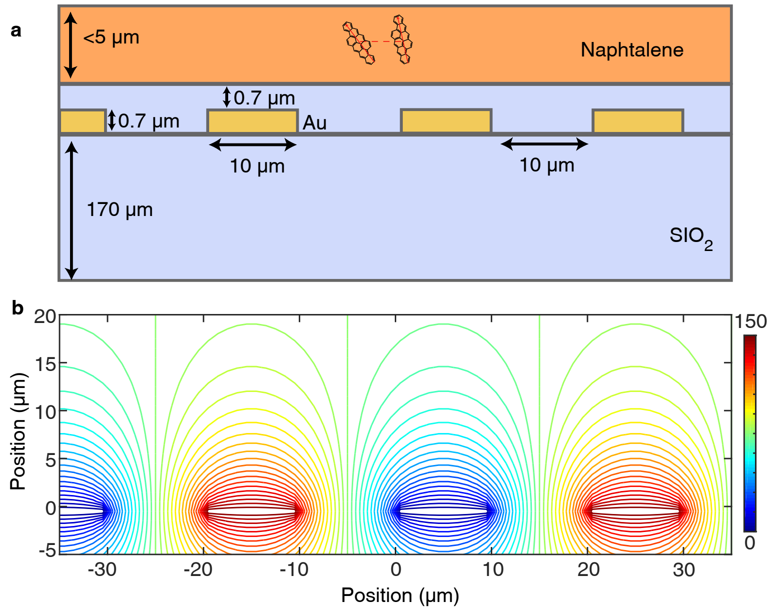

Supplementary Fig. 2: **Characteristics of the sample and electrodes.**
a, Schematic of the sample and electrodes. A comb of gold interdigitated electrodes with a spacing of 20 µm is sputtered onto a glass coverslip over which a 0.7 µm-thick SiO_2 layer is deposited by plasma-enhanced chemical vapor deposition. **b**, Distribution of electrical potential around the electrodes. In between the electrodes and for an applied voltage of 150 V, the electric field reaches a value of 25 MV m^{-1}, which is close to the breakdown value in silica[1].

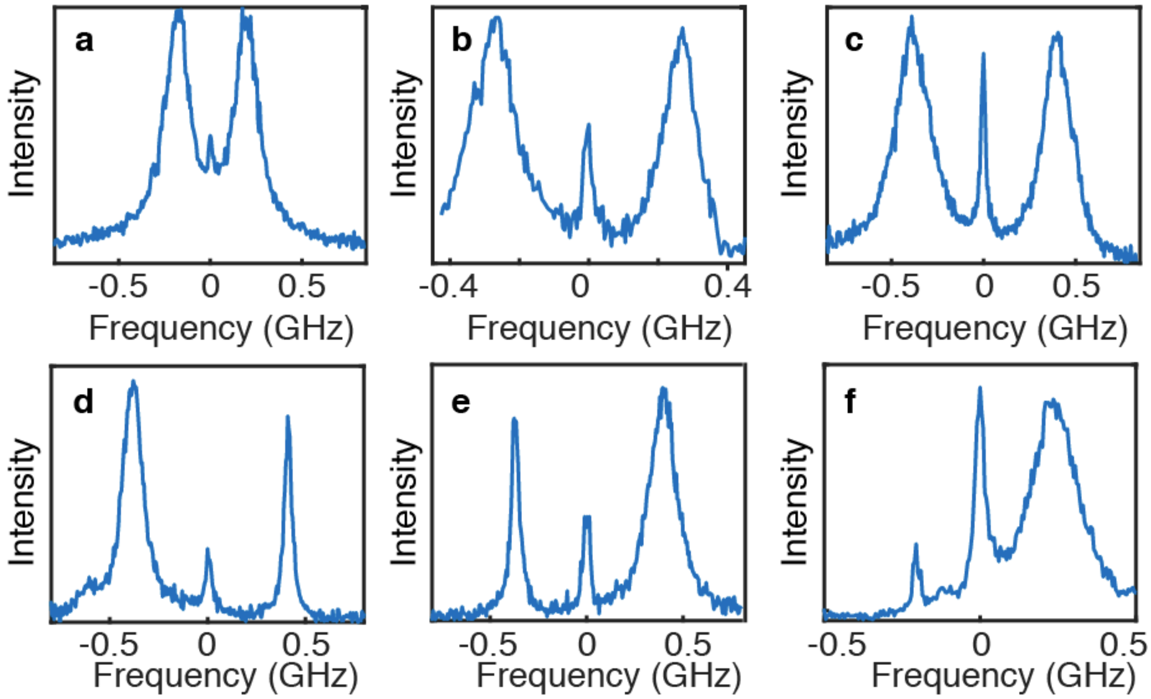

Supplementary Fig. 3: Excitation spectra of various pairs of coupled molecules.
a-f, Fluorescence excitation spectra recorded for six pairs of coupled molecules, recorded with an excitation intensity of ~ 100 W cm^{-2}. Each spectrum shows a sharp two-photon transition located midway between the symmetric and antisymmetric resonances. For each of these spectra, we have checked the quadratic dependence of the two-photon transition amplitude with the excitation intensity. These six examples show different coupling strengths and dipole orientations with J or H configurations.

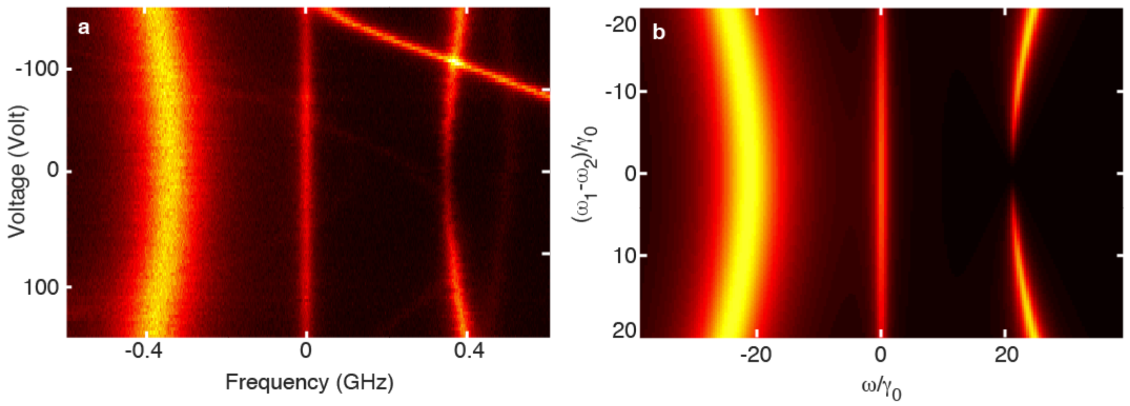

Supplementary Fig. 4: Molecular entanglement manipulated by Stark effect.
Experimental (**a**) and simulated (**b**) spectral trails of a pair of coupled molecules (the same as in Fig. 4), in the case of an excitation with a tightly focused Gaussian beam with an intensity of 220 W cm^{-2}. For the simulations, the molecular and laser interaction parameters are deduced from the analysis described in the Methods section. In **a**, one can notice the spectral trail of a third, uncoupled molecule, which crosses the other trails under the effect of a large Stark shift. This trail is discarded in the simulations in **b**.

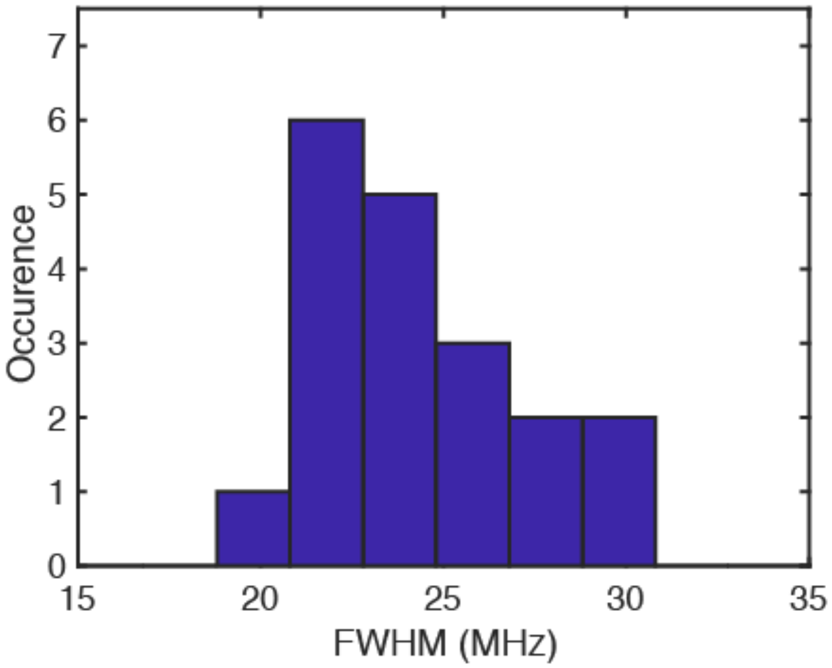

Supplementary Fig. 5: Distribution of the homogenous linewidths at 2K.
The histogram is built from the excitation spectra of 19 DBATT molecules recorded at an excitation intensity of 0.5 W cm^{-2}, which is well below the saturation intensity at 2 K. The centroid of this distribution gives $\gamma_0/2\pi = (23.0 \pm 4.5)$ MHz.

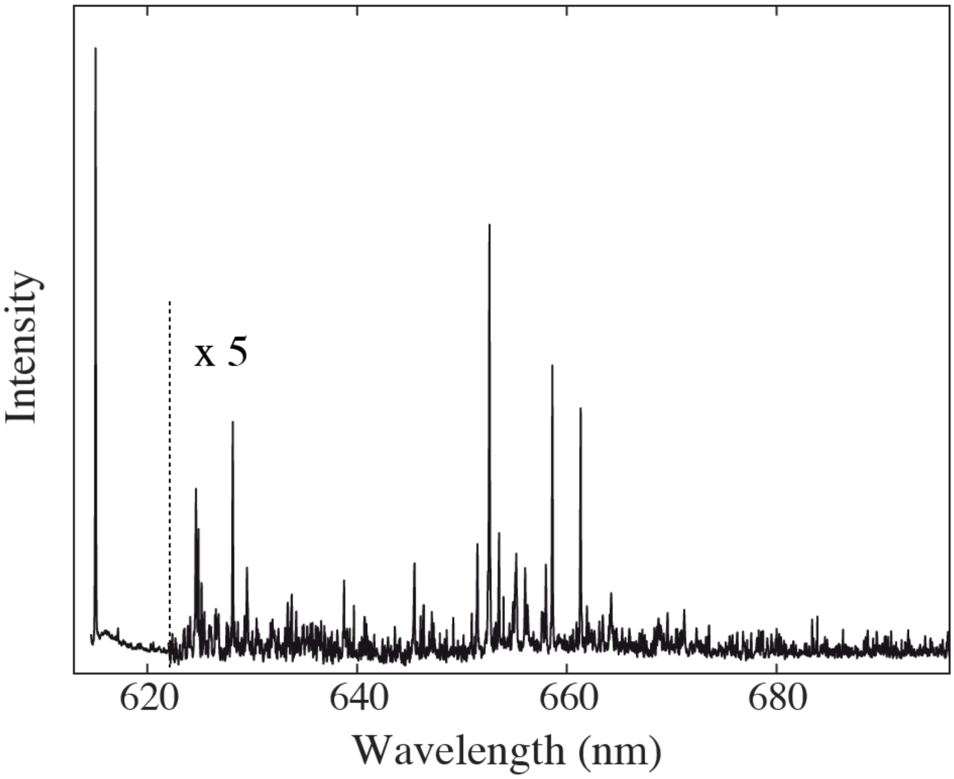

Supplementary Fig. 6: Determination of the combined Debye-Waller/Franck-Condon factor. Low-temperature emission spectrum of a single DBATT molecule in a naphthalene crystal, recorded over 60 s under an excitation at 605 nm with intensity 40 W cm^{-2}. It comprises a narrow and intense ZPL, vibrational fluorescence lines, and phonon sidebands. The relative weight of the ZPL in the entire emission spectrum is 0.3, which gives estimation for the combined Debye-Waller/Franck-Condon factor.

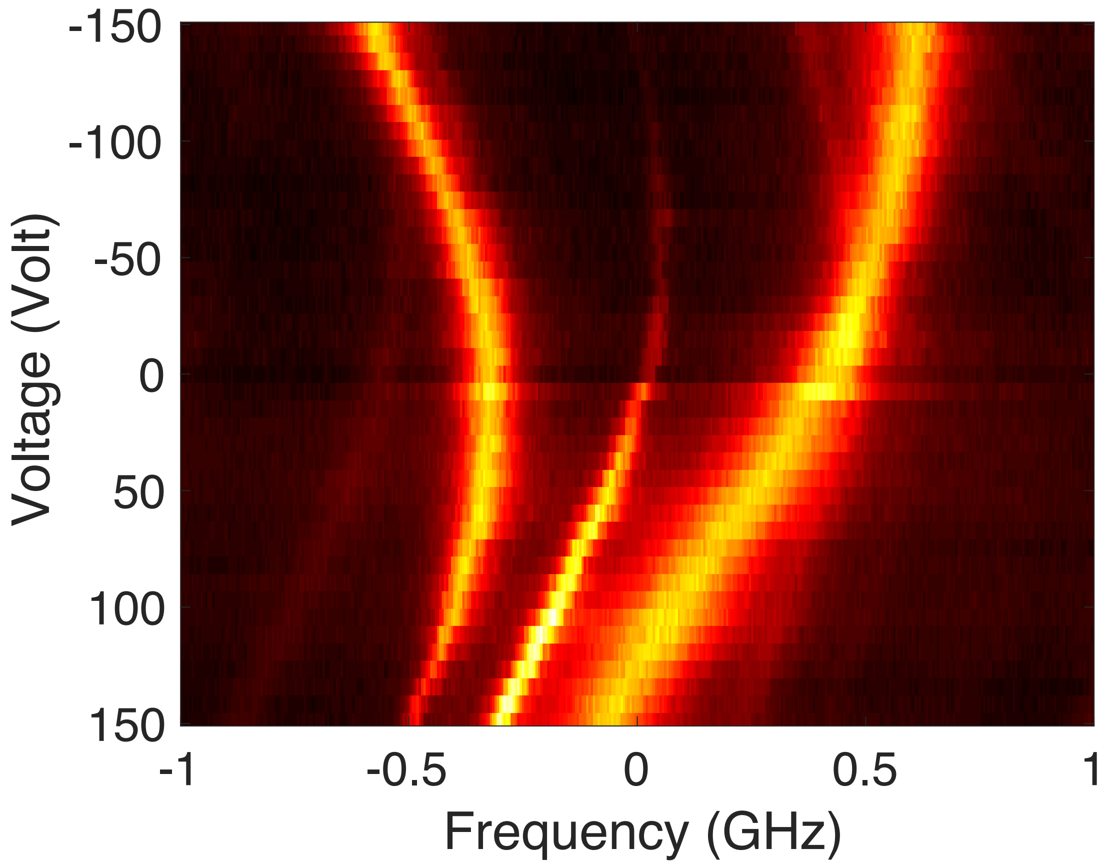

Supplementary Fig. 7: Raw Stark-tuned spectral trails.
These spectral trails are built from the raw fluorescence excitation spectra measured on the coupled molecules presented in Fig. 2f. They display quadratic and linear Stark shifts of the molecular resonances, leading to a net detuning of the molecular resonances and signatures of a variation of the degree of entanglement in the superradiant and subradiant states.

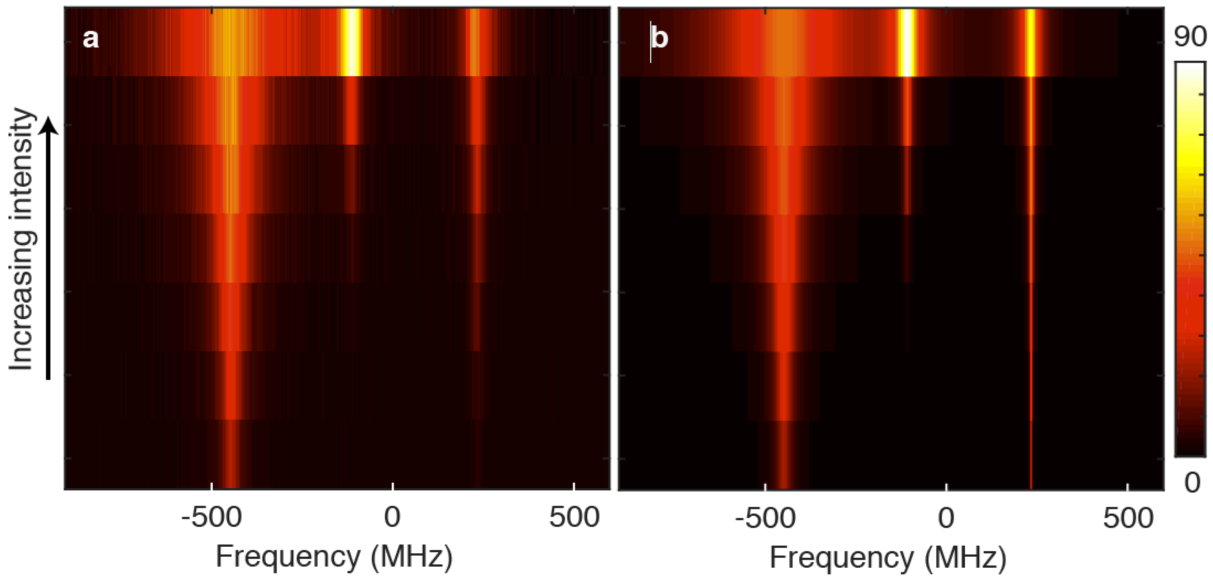

Supplementary Fig. 8: Intensity dependence of the excitation spectra.
a, Series of experimental excitation spectra recorded with a TEM_{00} excitation beam for increasing laser intensities from 2 W cm^{-2} to 250 W cm^{-2}, for the pair of coupled molecules presented in Fig. 4 and Fig. 1b. **b**, Spectra computed from the solutions of the master equation, using identical Rabi frequencies ($\Omega_1 = \Omega_2$). They yield $\gamma_0/2\pi = 21.5$ MHz, $V = -17\,\gamma_0$, $\Delta = 10\,\gamma_0$, $\gamma_{12} = 0.3\,\gamma_0$, and $I_{sat} = 40$ W cm^{-2}.

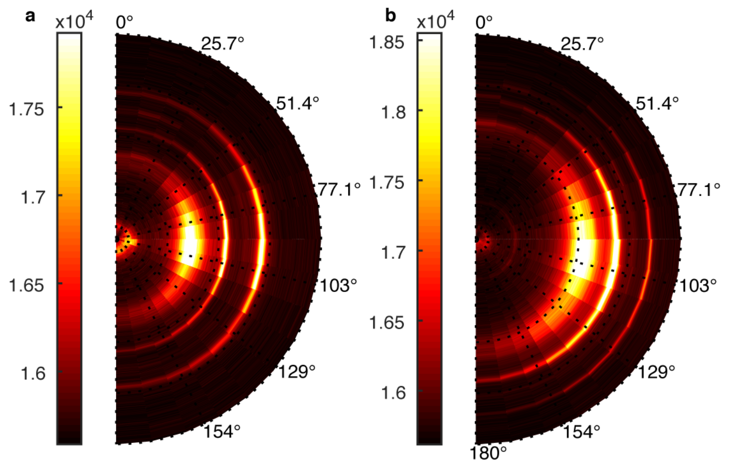

Supplementary Fig. 9: Determination of the dipole orientations in the focal plane. Series of excitation spectra recorded under a Gaussian-shaped laser beam with intensity 160 W cm^{-2}, for various angles of the polarizer placed in the detection path. In these fan-like charts, the radius represents the scanned frequency range, with increasing radii associated to increasing frequencies. **a**, Situation of weakly entangled emitters, under a voltage of 150 V. **b**, Situation of strongly entangled emitters, in zero voltage. In both cases, the resonances of the symmetric and antisymmetric states have a maximal intensity at the same polarizer angle (90°), which indicates that the in-plane dipole projections of the molecules are nearly parallel, within 5°.

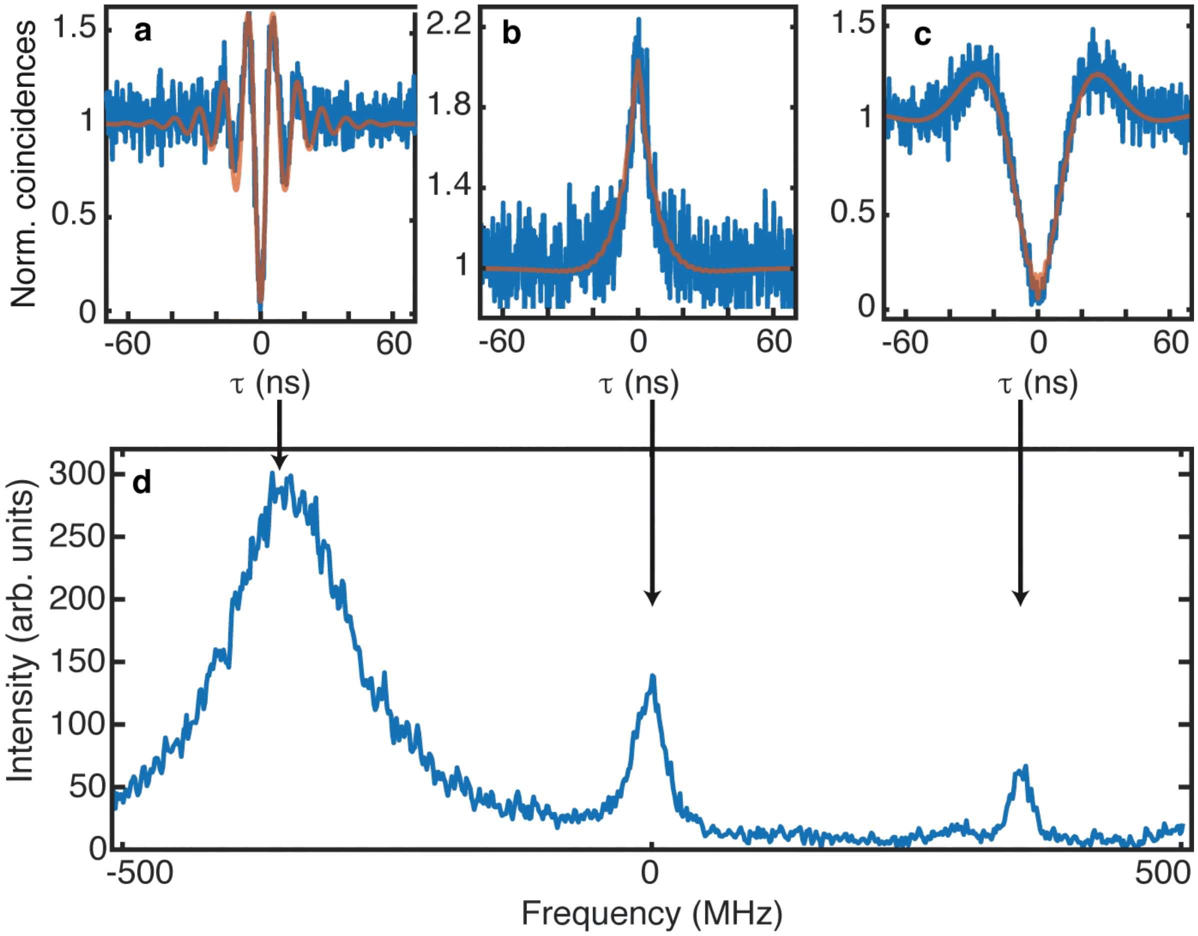

Supplementary Fig. 10: Photon coincidence histograms under selective excitation of the superradiant, subradiant and doubly excited states.

a,**b**,**c**, Normalized photon coincidence histograms measured on the pair of coupled molecules presented in Fig. 4 when the Gaussian-shaped laser beam of intensity 200 W cm^{-2} is tuned at resonance with the symmetric state (**a**), the two-photon transition (**b**) and the antisymmetric state (**c**). Photon bunching is evidenced in (**c**) as a signature of two-photon emission from the doubly excited state $|E\rangle$. The second-order correlation functions (red curves) are computed for nearly parallel dipoles in the J-configuration, $V = -17\,\gamma_0$, $\gamma_{12} = 0.3\,\gamma_0$, and $\Delta = 10\,\gamma_0$. The black arrows indicate the correspondence between the coincidence histograms and the selected ZPLs in the fluorescence excitation spectrum (**d**) recorded at the same intensity.

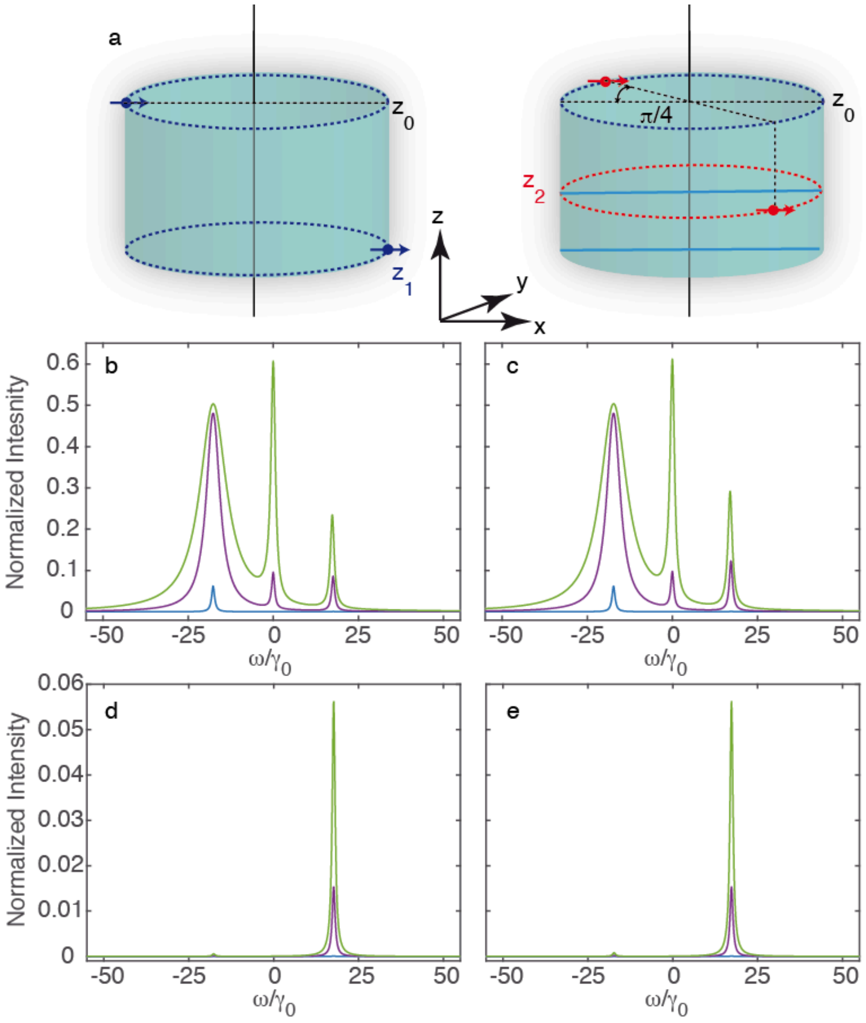

Supplementary Fig. 11: Tailoring the selection rules by laser field structuration.
a, Schematics of two sets of 3D locations of coupled molecules with parallel dipoles (along the x-axis) leading to the same coupling strength $V = -17\,\gamma_0$. Configuration 1 (left scheme): One molecule is located at x = -9 nm, y = 0 nm (coordinates in the focal plane) and z_0 = 0 nm (out-of-plane component), while the other is located at x = 9 nm, y = 0 nm and z_1 = -13.4 nm. Configuration 2 (right scheme): One molecule is located at x= -6.4 nm, y= 6.4 nm and z_0 = 0 nm, while the other is located at x = 6.4 nm, y = -6.4 nm, and z_2 = -1.3 nm. The in-plane separation distance between the coupled molecules is 18 nm in both configurations. The fluorescence excitation spectra are computed in the cases of an illumination with a Gaussian (**b**,**c**) or a doughnut (**d**,**e**) beam shape, taking a detuning $\Delta = 10\,\gamma_0$ and a cross damping rate $\gamma_{12} = 0.35\,\gamma_0$, i.e. α = 0.35. They are presented for three different excitation intensities: $6{\times}10^{-2}\,\mathrm{I}_s$ (blue curves), $4\,\mathrm{I}_s$(purple curves) and $16\,\mathrm{I}_s$ (green curves). The spectra in **b**,**d** (resp. **c**,**e**) correspond to the configuration 1 (resp. 2) depicted in **a**.

Supplementary Note 1 : Eigen energies of the molecular system and steady state populations of $|G\rangle$, $|S\rangle$, $|A\rangle$ and $|E\rangle$

Starting from the Hamiltonian H specified in equation (1), which is valid in the rotating wave approximation, we perform the unitary transformation $\mathrm{U} = e^{i\omega_{\mathrm{L}}t(|E\rangle\langle E|-|G\rangle\langle G|)}$ to remove the explicit temporal dependence of the driving field. In this basis, the Hamiltonian $\breve{\mathrm{H}}$ writes :

$$\breve{\mathrm{H}} = \mathrm{UHU}^{\dagger} + i\hbar\frac{\partial \mathrm{U}}{\partial t}\mathrm{U}^{\dagger}$$

where $\mathrm{U}^{\dagger}$ denotes the Hermitian adjoint of U, which leads to the expression:

$$\begin{aligned}\breve{\mathrm{H}} = {}& \hbar(\omega_0 - \omega_{\mathrm{L}})(|E\rangle\langle E| - |G\rangle\langle G|) + \hbar\Lambda(|S\rangle\langle S| - |A\rangle\langle A|) \\ & - \frac{\hbar}{2}\{[(a\Omega_1 + b\Omega_2)|E\rangle\langle S| + (b\Omega_1 + a\Omega_2)|S\rangle\langle G|] \\ & + [(a\Omega_2 - b\Omega_1)|E\rangle\langle A| - (b\Omega_2 - a\Omega_1)|A\rangle\langle G|] + h.c.\}\end{aligned}$$

In the following we assume that the two emitters are maximally entangled (i.e. $V = \Lambda$) and the laser frequency ω_{L} is set to ω_0.

When the focused laser spot is swept along the inter-emitter (x) axis, both Rabi frequencies $\Omega_1$and Ω_2 are real numbers and the diagonalization of $\breve{\mathrm{H}}$ is straightforward, with eigen-energies are given by :

$$\begin{cases} \mathrm{E}_1 = \frac{\hbar}{2}\left(\Lambda - \sqrt{(\Omega_1 + \Omega_2)^2 + \Lambda^2}\right) \\ \mathrm{E}_2 = -\frac{\hbar}{2}\left(\Lambda + \sqrt{(\Omega_1 - \Omega_2)^2 + \Lambda^2}\right) \\ \mathrm{E}_3 = \frac{\hbar}{2}\left(\Lambda + \sqrt{(\Omega_1 + \Omega_2)^2 + \Lambda^2}\right) \\ \mathrm{E}_4 = -\frac{\hbar}{2}\left(\Lambda - \sqrt{(\Omega_1 - \Omega_2)^2 + \Lambda^2}\right) \end{cases},$$

and the corresponding eigen-vectors by

$$\begin{cases} |1\rangle = N_1\left[|E\rangle + \frac{\sqrt{2}(\Omega_1+\Omega_2)}{\Lambda+\sqrt{(\Omega_1+\Omega_2)^2+\Lambda^2}}|S\rangle + |G\rangle\right] \\ |2\rangle = N_2\left[-|E\rangle + \frac{\sqrt{2}(\Omega_2-\Omega_1)}{\Lambda-\sqrt{(\Omega_1-\Omega_2)^2+\Lambda^2}}|A\rangle + |G\rangle\right] \\ |3\rangle = N_3\left[|E\rangle + \frac{\sqrt{2}(\Omega_1+\Omega_2)}{\Lambda-\sqrt{(\Omega_1+\Omega_2)^2+\Lambda^2}}|S\rangle + |G\rangle\right] \\ |4\rangle = N_4\left[-|E\rangle + \frac{\sqrt{2}(\Omega_2-\Omega_1)}{\Lambda+\sqrt{(\Omega_1-\Omega_2)^2+\Lambda^2}}|A\rangle + |G\rangle\right] \end{cases},$$

where N_{i} (i = 1,2,3,4) are normalization coefficients:

$$N_1 = \left[2 + \frac{2(\Omega_1+\Omega_2)^2}{\left(\Lambda+\sqrt{(\Omega_1+\Omega_2)^2+\Lambda^2}\right)^2}\right]^{-\frac{1}{2}},\ N_2 = \left[2 + \frac{2(\Omega_2-\Omega_1)^2}{\left(\Lambda-\sqrt{(\Omega_1-\Omega_2)^2+\Lambda^2}\right)^2}\right]^{-\frac{1}{2}}$$

$$N_3 = \left[2 + \frac{2(\Omega_1+\Omega_2)^2}{\left(\Lambda-\sqrt{(\Omega_1+\Omega_2)^2+\Lambda^2}\right)^2}\right]^{-\frac{1}{2}},\ N_4 = \left[2 + \frac{2(\Omega_2-\Omega_1)^2}{\left(\Lambda+\sqrt{(\Omega_1-\Omega_2)^2+\Lambda^2}\right)^2}\right]^{-\frac{1}{2}}.$$

When the focused laser spot is swept along the orthogonal direction (y-axis), the two Rabi frequencies are complex numbers with identical modulus and different phases: $\Omega_2 = \Omega_1 e^{-i\varphi}$, with Ω_1 real. For a laser beam with a Laguerre Gaussian mode LG_{01}, this phase difference φ corresponds to the angle separating the segments stemming from the doughnut center and connecting each emitter (see Fig. 5a). The eigenenergies E are solutions of the following polynom obtained by diagonalization of the Hamiltonian expressed in the $\{|g_1, g_2\rangle, |g_1, e_2\rangle, |e_1, g_2\rangle, |e_1, e_2\rangle,\}$ basis [2]

$$E^4 - E^2\left(\Lambda^2 + {\Omega_1}^2\right) - \Lambda {\Omega_1}^2 \cos(\varphi) E = 0$$

The solution $E = 0$ corresponds to the ground state energy and does not depends on y. Note that two of the four eigen-energies become degenerate when the center of the doughnut beam is located at one of the two specific positions where $\varphi = \pm\frac{\pi}{2}$. This results in a dip in the fluorescence image due to destructive interference between the two excitation paths from $|G\rangle$ to $|E\rangle$. The eigen-energies are in this case:

$$\begin{cases} E_{G'} = 0 \\ E_{E'} = 0 \\ E_{S'} = \sqrt{V^2 + {\Omega_1}^2} \\ E_{A'} = -\sqrt{V^2 + {\Omega_1}^2} \end{cases}$$

For any other position of the beam along the y-axis, the derivation of the steady state populations of $|G\rangle$, $|S\rangle$, $|A\rangle$ and $|E\rangle$ is done numerically.

Supplementary Note 2 : ESSat images of coupled or uncoupled emitters.

In the following, we consider two coupled molecules having identical resonance frequencies ω_0 and emission rates γ_0, located 60 nm apart in the x-direction with parallel dipoles oriented along the y-axis (H-type dipole configuration). We use the combined Debye-Waller/Franck-Condon factor α to take into account the presence of vibrational states of the electronic ground state of the molecules.

The ESSat images are obtained by computing the fluorescence signal proportional to $2\rho_{e,e} + \rho_{e,g} + \rho_{g,e}$ for each position of the doughnut beam, where $\rho_{e,e}$, $\rho_{e,g}$, $\rho_{g,e}$ are the steady state populations of $|ee\rangle$, $|eg\rangle$, and $|ge\rangle$ derived from the master equation for the density operator ρ [3]:

$$\dot{\rho} = \frac{1}{i\hbar}[\mathcal{H}, \rho] - \frac{1}{2}\sum_{i,j=1,2} \Gamma_{ij}\,(S_i^+ S_j^- \rho + \rho S_i^+ S_j^- - 2S_j^- \rho S_i^+)$$

where the first term describes the coherent evolution of the density matrix and the second term the dissipative contribution, with the following notations :

- $\Gamma_{12} = \Gamma_{21}$ is the cross damping rate and $\Gamma_{11} = \Gamma_{22} = \gamma_0$ is the radiative decay rate of a molecule,
- $\mathcal{H} = \mathcal{H}_0 + \mathcal{H}_{dd} + \mathcal{H}_{laser}$ is the total Hamiltonian of the system,
- $\mathcal{H}_0 = \hbar\omega_1 S_1^+ S_1^- + \hbar\omega_2 S_2^+ S_2^-$ is the Hamiltonian of the two bare molecules,
- $\mathcal{H}_{d-d} = \sum_{i,j,i\neq j}\left(V S_i^+ S_j^- + h.c.\right)$ is the Hamiltonian of the non retarded dipole-dipole interaction with an amplitude V (valid for a separation distance between molecules less than the optical wavelength),
- $\mathcal{H}_{laser} = -\frac{1}{2}\hbar[\Omega_1 S_1^+ + \Omega_2 S_2^+ + h.c.]$ is the interaction between the two emitters and the coherent laser field. Neglecting the vectorial nature of light, we use the following in-plane distribution of electric field, expressed in polar coordinates, to compute the two Rabi frequencies Ω_1 and Ω_2 :

$$E(r,\varphi) = E_0\left(\frac{\sqrt{e}r}{r_0}\right)e^{-\frac{r^2}{2r_0^2}}e^{i\varphi}$$

We first consider uncoupled molecules by setting $\alpha = 0$ (Supplementary Fig. 12). At low intensity ($5\,I_s$), where I_s is the saturation intensity of a molecule, and for a laser frequency set to ω_0 , we observe a single fluorescence dip centered between the two emitters without super resolving them (Supplementary Fig. 12a). When increasing the excitation intensity, the fluorescence dip splits into two minima centered on each emitter (Supplementary Figs. 12b-d). At high saturations, the amplitudes of these dips reach half of the maximal signal, as expected when one molecule is excited while the other one is not.

In the case of coupled molecules ($\alpha = 1$), this picture drastically changes. Supplementary Fig. 13 shows ESSat images of a coupled pair for various laser intensities ranging from $5\,I_s$ to $8.2 \times 10^5 I_s$. As in the case of uncoupled molecules, the ESSat image fails to super-resolve the emitters at low excitation intensities (Supplementary Fig. 13a). For intermediate intensities (Supplementary Fig. 13b), two fluorescence dips show up along the y-axis (the direction orthogonal to the segment connecting the two emitters). These dips correspond to destructive interference between the two excitation pathways $|G\rangle \longrightarrow |A\rangle \rightarrow |E\rangle$ and $|G\rangle \longrightarrow |S\rangle \rightarrow |E\rangle$, which occurs at the two locations where $\Omega_2 = \Omega_1 e^{\pm i\frac{\pi}{2}}$. Indeed, the two-photon transition has a vanishing effective Rabi frequency:

$$\hbar\Omega_{EG}^{\text{eff}} = \frac{\langle E|\mathrm{H}|A\rangle\langle A|\mathrm{H}|G\rangle}{\hbar(\omega_0-\omega+\Lambda)} + \frac{\langle E|\mathrm{H}|S\rangle\langle S|\mathrm{H}|G\rangle}{\hbar(\omega_0-\omega-\Lambda)} = \frac{\hbar\Omega_1^2(\pm ia-b)(a\mp ib)}{4\Lambda} - \frac{\hbar\Omega_1^2(a\pm ib)(\pm ia+b)}{4\Lambda} = 0.$$

Upon increasing intensities, a structured fluorescence landscape develops along x, with the onset of two additional dips at high saturation, as shown in Supplementary Fig. 10c,d. In the regime of extremely high saturations, four dips appear along the x-axis (Supplementary Fig. 13e,f). The positions of the fluorescence minima correspond to the locations where two eigenenergies in the Hamiltonian $\mathcal{H}$ become degenerate (Fig. 5d and Supplementary Note 1).

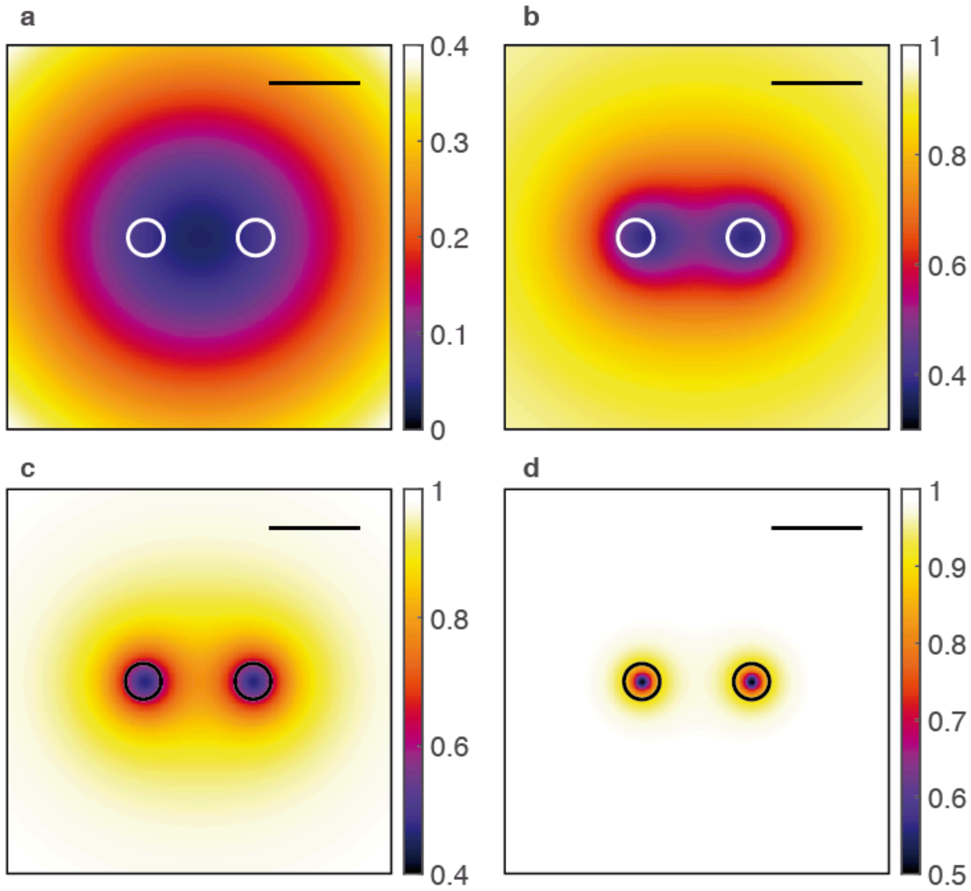

Supplementary Fig. 12: Simulated ESSat images of two identical, uncoupled molecules. The fluorescence images are computed for an excitation with a resonant doughnut-shaped laser beam, for four different excitation intensities: $5\,I_s$ (**a**), $130\,I_s$ (**b**), $2025\,I_s$ (**c**), and $4.6 \times 10^3\,I_s$ (**d**). The circles mark the positions of the emitters. The fluorescence signal is coded with a color bar and normalized to twice the maximal signal stemming from a single molecule. The scale bars are 50 nm.

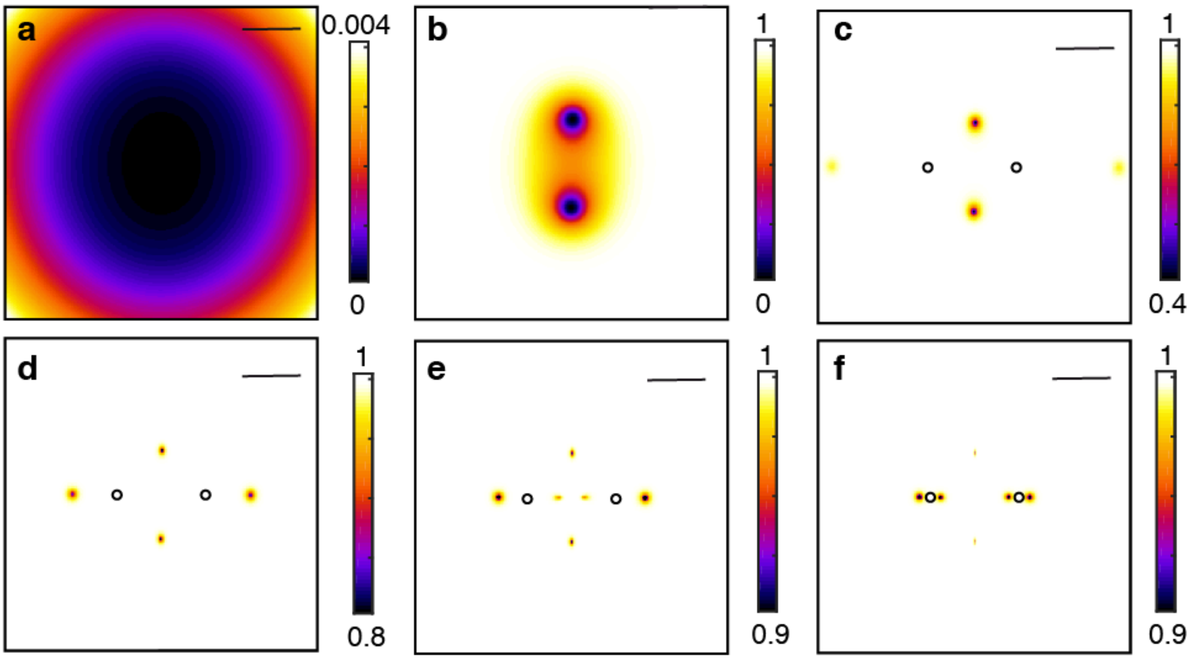

Supplementary Fig. 13: Computed ESSat images of pair of identical, coupled molecules.

The coupled pair is characterized by the following parameters: $\Delta = 2\,\gamma_0$, $V = -22\,\gamma_0$, $\alpha = 1$ and $\gamma_{12} = \gamma_0$. The ESSat fluorescence images are computed for an excitation with a resonant doughnut-shaped laser beam, for five different excitation intensities: $5\,I_s$ (**a**), $2000\,I_s$ (**b**), $1.3{\times}10^4\,I_s$ (**c**), $1.2{\times}10^5\,I_s$ (**d**) $4.5{\times}10^5\,I_s$ (**e**) and $8.2{\times}10^5\,I_s$ (**f**). The circles mark the positions of the emitters. The fluorescence signal is coded with a color bar and normalized to its maximal value. The scalebars are 50 nm.

References :

1. Klein, N. & Gafni, H. The Maximum Dielectric Strength of Thin Silicon Oxide Films. *IEEE Transactions on Electron Devices* **13,** 281–289 (1966).
2. Varada, G. & Agarwal, G. Two-photon resonance induced by the dipole-dipole interaction. *Phys. Rev. A* **45,** 6721–6729 (1992).
3. Ficek, Z. & Swain, S. *Quantum Interference and Coherence : Theory and Experiments*. (Springer, 2014).
